# Effect of LncRNA LOC106505926 on myogenesis and Lipogenesis of porcine primary cells

**DOI:** 10.1186/s12864-024-10422-y

**Published:** 2024-05-30

**Authors:** Mingyue Shi, Shuai Yang, Xiaolei Zhao, Di Sun, Yifei Li, Jingxian Yang, Meng Li, Chunbo Cai, Xiaohong Guo, Bugao Li, Chang Lu, Guoqing Cao

**Affiliations:** 1 College of Animal Science, Shanxi Agricultural University, Taigu, 030801 China; 2 Shanxi Animal Husbandry Technology Extension Service Center, Taiyuan, 030001 China

**Keywords:** Pig, LOC106505926, Skeletal muscle satellite cells, Preadipocytes, Myogenesis, Lipogenesis

## Abstract

**Background:**

Skeletal muscle development and fat deposition have important effects on meat quality. The study of regulating skeletal muscle development and fat deposition is of great significance in improving the quality of carcass and meat. In the present study, whole transcriptome sequencing (including RNA-Seq and miRNA-Seq) was performed on the longissimus dorsi muscle (LDM) of Jinfen White pigs at 1, 90, and 180 days of age.

**Results:**

The results showed that a total of 245 differentially expressed miRNAs were screened in any two comparisons, which may be involved in the regulation of myogenesis. Among them, compared with 1-day-old group, miR-22-5p was significantly up-regulated in 90-day-old group and 180-day-old group. Functional studies demonstrated that miR-22-5p inhibited the proliferation and differentiation of porcine skeletal muscle satellite cells (PSCs). Pearson correlation coefficient analysis showed that long non-coding RNA (lncRNA) LOC106505926 and *CXXC*5 gene had strong negative correlations with miR-22-5p. The LOC106505926 and *CXXC*5 were proven to promote the proliferation and differentiation of PSCs, as opposed to miR-22-5p. In terms of mechanism, LOC106505926 functions as a molecular sponge of miR-22-5p to modulate the expression of *CXXC*5, thereby inhibits the differentiation of PSCs. In addition, LOC106505926 regulates the differentiation of porcine preadipocytes through direct binding with FASN.

**Conclusions:**

Collectively, our results highlight the multifaceted regulatory role of LOC106505926 in controlling skeletal muscle and adipose tissue development in pigs and provide new targets for improving the quality of livestock products by regulating skeletal muscle development and fat deposition.

**Supplementary Information:**

The online version contains supplementary material available at 10.1186/s12864-024-10422-y.

## Introduction

Pork is the main source of meat for the Chinese people. Skeletal muscle accounts for about 40% of the body weight and 50-75% of all body proteins [1]. The characteristics of skeletal muscle also directly affect pork quality. Muscle fiber numbers are already established in the embryonic period [2]. Muscle development after birth mainly depends on the proliferation and differentiation of skeletal muscle satellite cells [3, 4], which are muscle tissue stem cells located between the muscle fiber substrate and the plasma coat [5]. Skeletal muscle satellite cells are activated in response to muscle injury and undergo cell proliferation, differentiation, and fusion to form new myotubes [6]. Skeletal muscle development is a complex process, current studies have found that many transcription factors are involved in the regulation of myogenesis, such as myogenic regulatory factors (MRFs) including *Myf*5, *MyoD*, *MyoG*, *Myf*6, myocyte enhancer factor-2 (MEF-2) family [7–9] and Pax family members [10, 11]. Recent studies have found that, in addition to transcription factors, non-coding RNAs are also involved in the regulation of muscle development, including miRNAs and lncRNAs.

Fat deposition is also an important biological process that affects the quality and growth efficiency of pork. Adipocytes originate from the embryonic mesoderm and are formed by the differentiation of preadipocytes. The transformation of preadipocytes to mature adipocytes determines the process of fat deposition [12]. Peroxisome proliferator-activated receptor γ (PPARγ) [13], fatty acid synthase (FASN), fatty acid binding protein 4 (FABP4) [14] and many key regulators of adipogenic differentiation play an important role in adipocyte differentiation and deposition. Many lncRNAs have been found to play a role in adipocyte thermogenesis [15, 16], adipocyte metabolism [17] and adipocyte differentiation [18].

Long non-coding RNA (lncRNA) is a kind of linear RNA with a length of more than 200 nt, which has no protein coding ability and has a wide subcellular distribution in cells [19]. This wide subcellular distribution determines the diversity of its functional mechanism. The mechanism of lncRNAs acting as ceRNAs has attracted much attention [20], but lncRNAs also have other mechanisms that play an important role in a variety of biological processes. H19 is one of the most known lncRNAs, and it plays a key role in the differentiation of skeletal muscle [21, 22]. By competitively binding to miR-140-5p, H19 inhibits the differentiation of skeletal muscle satellite cells in porcine [23]. In vivo, overexpression of H19 improved insulin sensitivity and mitochondrial biogenesis, silencing H19 impaired adipogenesis, oxidative metabolism and mitochondrial respiration in brown adipocytes [24]. Previous studies have found an obesity-related lncRNA lnc-ORA, which had a seven-fold higher expression in ob/ob mice than WT mice. Lnc-ORA knockdown inhibits adipocyte differentiation by regulating the PI3K/AKT/mTOR signaling pathway [25]. In addition, lnc-ORA was found to play a role in myogenesis. It can inhibit skeletal muscle myogenesis and reduce the stability of myogenic genes by acting as a sponge for miR-532-3p or interacting with insulin-like growth factor 2 mRNA binding protein 2 [26]. These indicate that lncRNA plays a role in a variety of biological processes through different mechanisms and lncRNA is indispensable in the development of various tissues.

In the present research, miRNA-Seq was performed on longissimus dorsi muscle (LDM) of Jinfen White pigs at 1, 90 and 180 days of age to screen candidate miRNAs that can regulate porcine myogenesis. The LOC106505926/miR-22-5p/*CXXC*5 regulatory network was constructed to regulate the proliferation and differentiation of PSCs. In addition, LOC106505926 was testified to inhibit the differentiation of preadipocytes by directly binding to FASN. This study may provide a molecular basis for understanding the development process of porcine skeletal muscle and adipose tissue.

## Results

### Candidate miRNAs regulating muscle development were screened by miRNA-seq

Whole transcriptome sequencing (including RNA-Seq and miRNA-Seq) was performed on LDM of Jinfen White pigs at 1, 90 and 180 days of age [27]. For miRNAs, on average, 14.45 million, 17.68 million and 15.07 million raw reads and 14.12 million, 17.44 million, 14.87 million clean reads were obtained from LDM of Jinfen White pigs at 1, 90 and 180 days of age respectively. In addition, the error rate of all sequencing data sets was less than 0.01%, the value of Q20 was above 98%, Q30 value was above 94% and the average GC content was 49%, Specific data are presented in the supplementary Table S1.

**Fig. 1:**
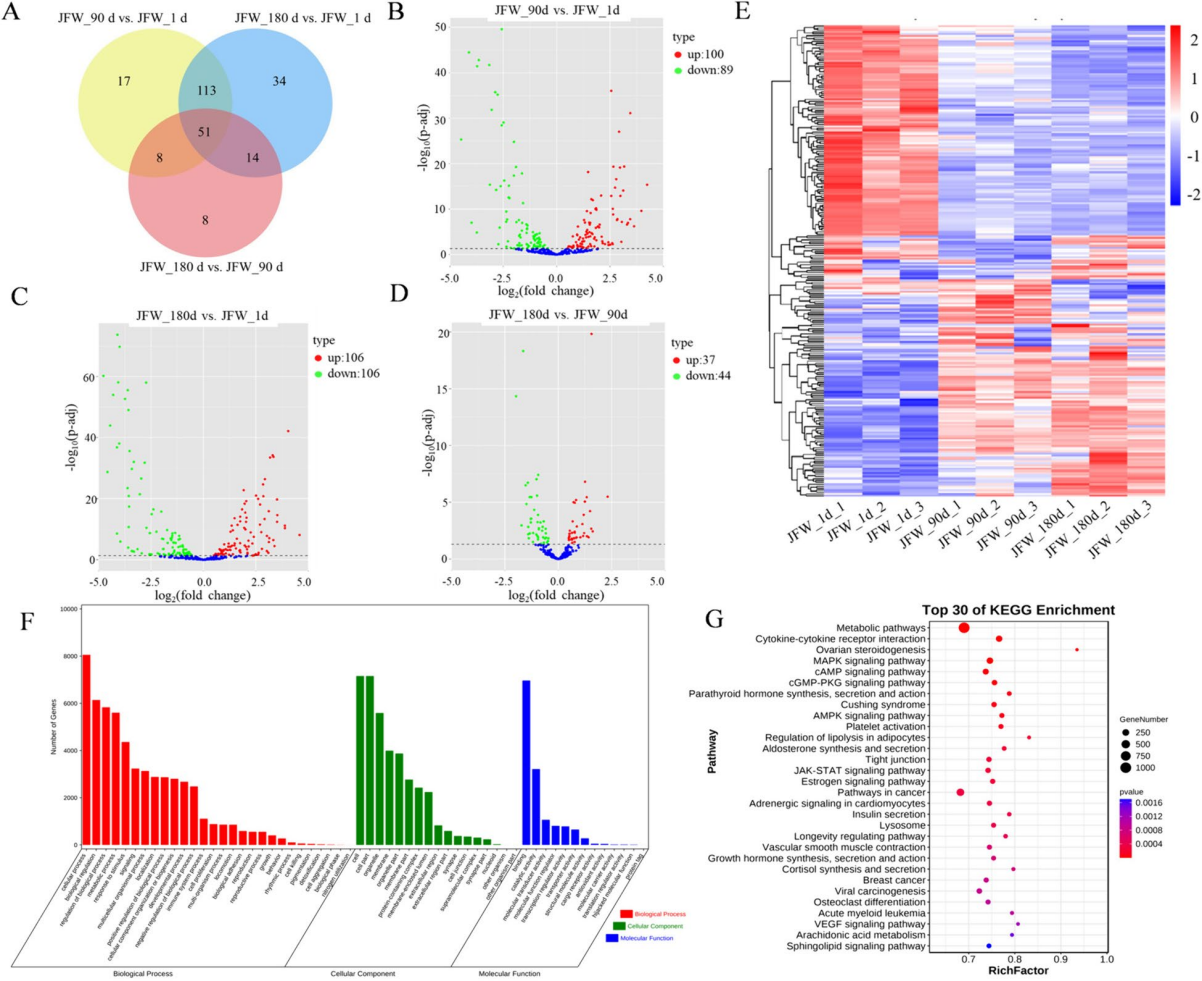
Candidate miRNAs regulating muscle development were screened. **A** Venn diagram of DE miRNAs. **B**-**D** Volcano plot of DE miRNAs. **E** Heat map of DE miRNAs. **F** GO enrichment results of DE miRNAs. **G** The results of KEGG analysis of DE miRNAs

Differentially expressed miRNAs (DE miRNAs) in any two comparisons were screened out (Fig. 1A). Specifically, 100 miRNAs were up-regulated and 89 miRNAs were down-regulated in 90-day-old muscle tissues compared with 1-day-old (Fig. 1B). There were 106 up-regulated and 106 down-regulated miRNAs in 180-day-old muscle tissues compared with 1-day-old (Fig. 1C). Compared with the 90-day-old group, there were 37 up-regulated miRNAs and 44 down-regulated miRNAs in the 180-day-old group (Fig. 1D). The heat map showed the DE miRNAs expression patterns at different days of age (Fig. 1E). GO and KEGG results showed that the target genes of DE miRNAs were mainly enriched in MAPK, AMPK, JAK-STAT, VEGF and other signaling pathways, which were related to muscle development (Fig. 1F and G). Thus, these DE miRNAs may be involved in the regulation of porcine myogenesis. Among DE miRNAs, miR-22-5p exhibited a notably higher fold change and displayed a strong negative regulatory association with target genes, and its target genes may play a role in the modulation of skeletal muscle development. Therefore, we focused on miR-22-5p for subsequent research.

### MiR-22-5p inhibits the proliferation and differentiation of PSCs

**Fig. 2:**
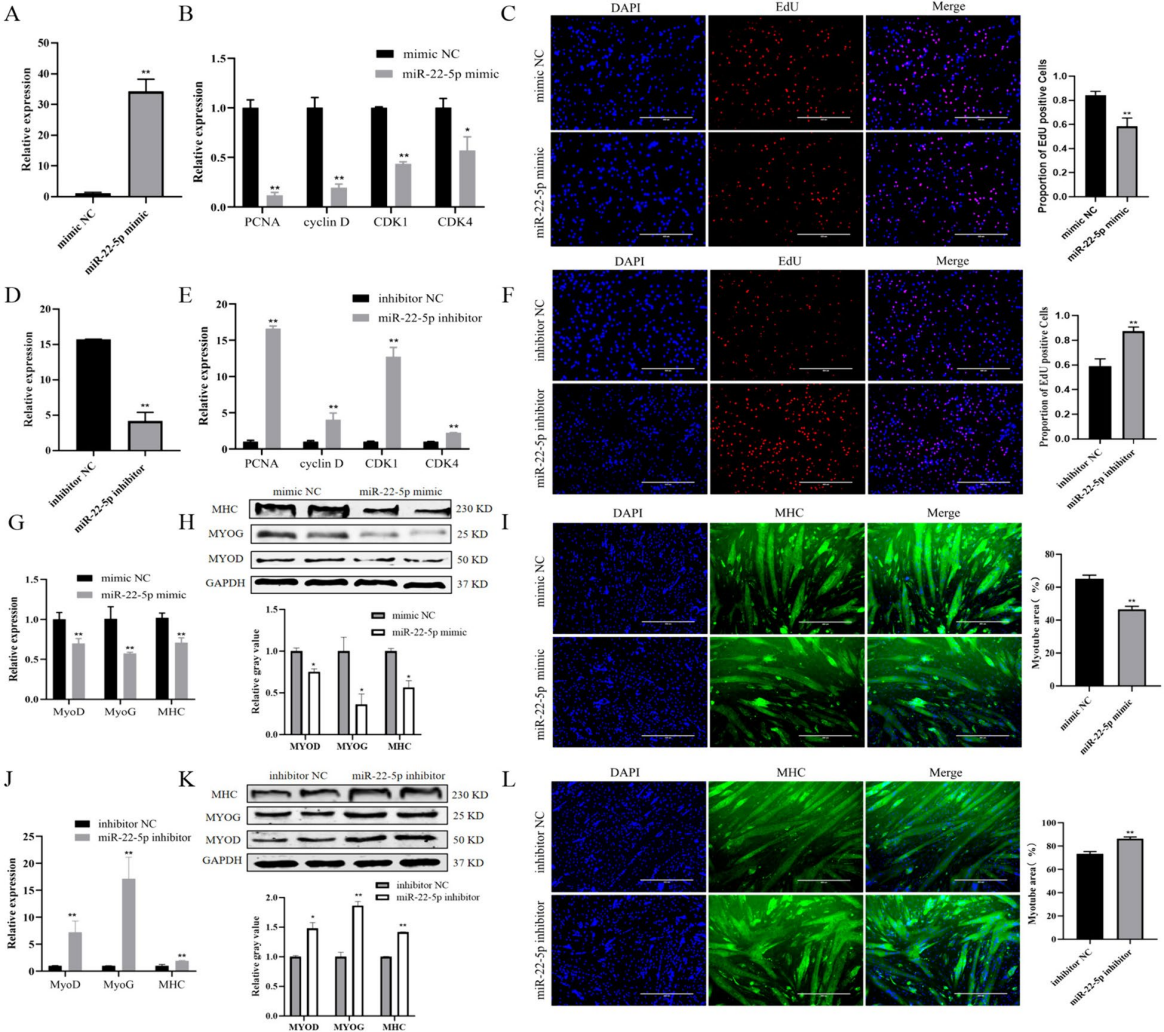
The effect of miR-22-5p on the proliferation and differentiation of PSCs. **A** Cell transfection efficiency of miR-22-5p mimic. **B** The expression changes of proliferation marker genes after transfected with miR-22-5p mimic. **C** The EdU results after transfected with miR-22-5p mimic. **D** Cell transfection efficiency of miR-22-5p inhibitor. **E** The expression changes of proliferation marker genes after transfected with miR-22-5p inhibitor. **F** The EdU results after transfected with miR-22-5p inhibitor. **G** The expression changes of differentiation marker genes after transfected with miR-22-5p mimic at mRNA level. **H** The expression changes of differentiation marker genes after transfected with miR-22-5p mimic at protein level. **I** The results of immunofluorescence after transfected with miR-22-5p mimic. **J** The expression changes of differentiation marker genes after transfected with miR-22-5p inhibitor at mRNA level. **K** The expression changes of differentiation marker genes after transfected with miR-22-5p inhibitor at protein level. **L** The results of immunofluorescence after transfected with miR-22-5p inhibitor. (Mean ± SD; *P < 0.05, **P < 0.01. The blots were cut prior to hybridisation with antibodies, and images of blots for all replicates were provided in the supplementary file 4)

To further study the function of miR-22-5p in PSCs, miR-22-5p was overexpressed in PSCs. As Fig. 2A showed, the expression of miR-22-5p was significantly increased following miR-22-5p mimic transfection (Fig. 2A, *P* < 0.01). Additionally, the expression of proliferation marker genes was significantly decreased after transfection with miR-22-5p mimic (Fig. 2B, *P* < 0.05), along with a notable decrease in the number of EdU positive cells (Fig. 2C, *P* < 0.01). Conversely, transfection with miR-22-5p inhibitor led to a significant decrease in miR-22-5p expression (Fig. 2D, *P* < 0.01), while there was a significant increase in the expression of proliferation marker genes (Fig. 2E, *P* < 0.01) and the number of EdU positive cells (Fig. 2F, *P* < 0.01). These results indicated that miR-22-5p inhibited the proliferation of PSCs.

To investigate the impact of miR-22-5p on the myogenic differentiation of PSCs, the expression changes of key myogenic factors were detected. The results revealed a significant decrease in the expression of *MyoG*, *MyoD*, and *MHC* upon transfection with miR-22-5p mimic (Fig. 2G, *P* < 0.01). Consistent increase of protein level was also observed which is measured by Western blot (Fig. 2H). The immunofluorescence staining results showed that the number of myotubes was significantly decreased in miR-22-5p mimic group (Fig. 2I, *P* < 0.01). Conversely, transfection with miR-22-5p inhibitor led to opposite outcomes (Fig. 2J-L). The above results proved that miR-22-5p suppresses the differentiation of PSCs.

### MiR-22-5p binds to the 3′UTR of *CXXC*5 and LOC106505926

**Fig. 3:**
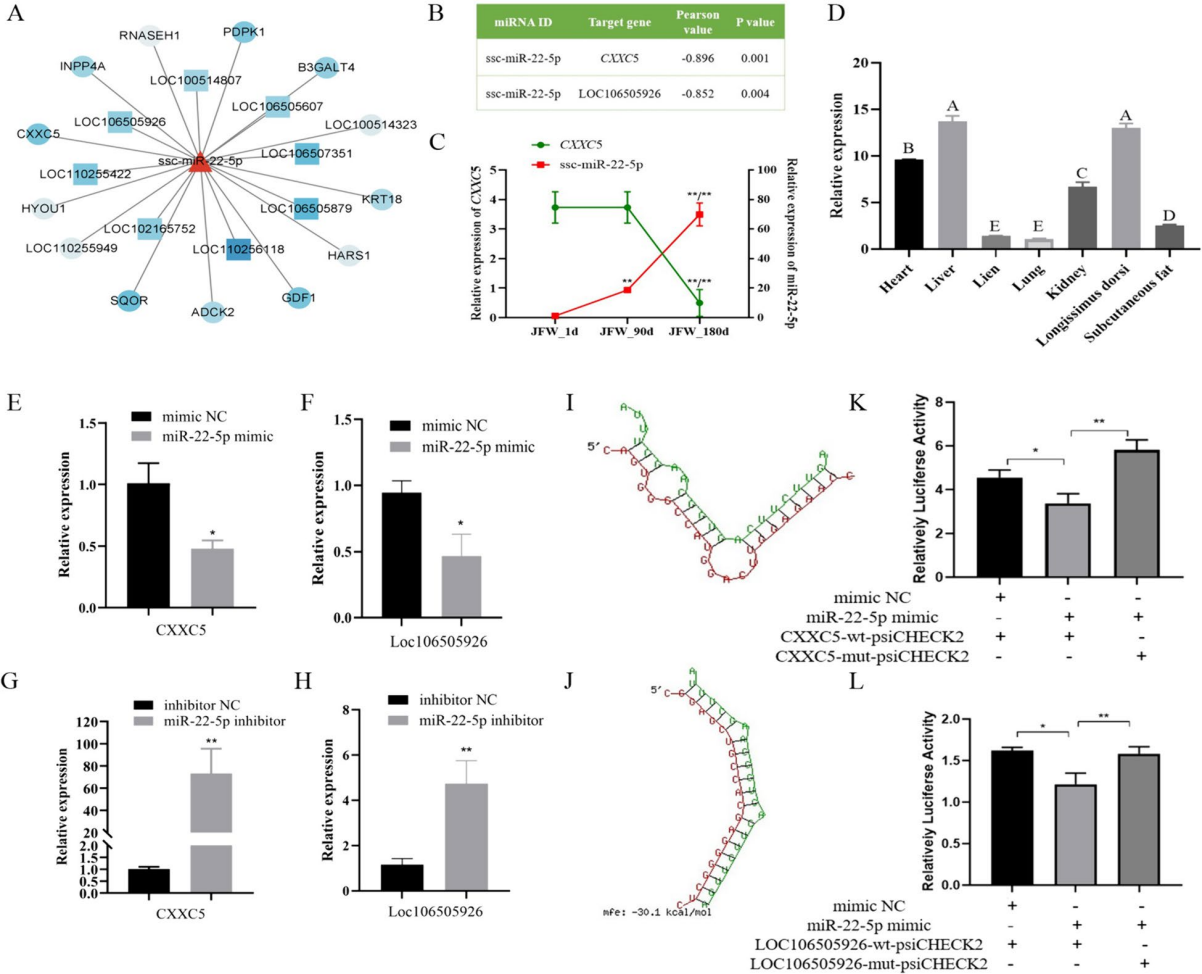
MiR-22-5p binds to the 3′UTR of *CXXC*5 and LOC106505926. **A** The correlation between miR-22-5p expression and its target genes. Blue from light to deep indicates that the pearson coefficient changes from 0 to -1. **B** The pearson coefficient between miR-22-5p and *CXXC*5, miR-22-5p and LOC106505926. **C** The expression levels of miR-22-5p and *CXXC*5 in LDM at three developmental stages. **D** Expression profiling of *CXXC*5. **E** Effect of miR-22-5p mimics transfection on relative expression of *CXXC*5. **F** Effect of miR-22-5p inhibitor transfection on relative expression of *CXXC*5. **G** Effect of miR-22-5p mimics transfection on relative expression of LOC106505926. **H** Effect of miR-22-5p inhibitor transfection on relative expression of LOC106505926. **I** RNAhybird predicts the binding of miR-22-5p to *CXXC*5-3′UTR. **J** RNAhybird predicts the binding of miR-22-5p to LOC106505926. **K** Dual-luciferase reporter assay verifies the binding of miR-22-5p to CXXC5-3′UTR. **L** Dual-luciferase reporter assay verifies the binding of miR-22-5p to LOC106505926. Different uppercase indicates extremely significant differences. (Mean ± SD; *P < 0.05, **P < 0.01. Values with different letters indicated significant differences)

Based on sequencing results, there were multiple mRNAs and lncRNAs associated with miR-22-5p. Among them,* CXXC*5 and LOC106505926 had a stronger negative correlation with miR-22-5p (Fig. 3A), with Pearson coefficient of -0.896 and − 0.852 (Fig. 3B). Expression patterns analysis found that the expression of miR-22-5p showed an upward trend with the increase of age, while *CXXC*5 expression showed a downward trend (Fig. 3C). Expression profiling revealed that *CXXC*5 had the highest expression in the liver and LDM (Fig. 3D, *P* < 0.01). The expression of *CXXC*5 and LOC106505926 was significantly decreased after transfection of miR-22-5p mimics (Fig. 3E, F, *P* < 0.05), while transfection of miR-22-5p inhibitor showed the opposite results (Fig. 3G, H). RNAhybird prediction showed that the 3′UTR of *CXXC*5 and LOC106505926 could bind to the seed sequence of miR-22-5p (Fig. 3I, J). The wild-type and mutant vectors of 3′UTR of *CXXC*5 and LOC106505926 were constructed respectively. The luciferase activity of miR-22-5p mimics and CXXC5-wt-psiCHECK2 group was significantly lower than other groups (Fig. 3K, *P* < 0.01), The luciferase activity of miR-22-5p mimics and LOC106505926-wt-psiCHECK2 group was also significantly lower than other groups (Fig. 3L, *P* < 0.01), indicating that miR-22-5p can bind to 3′UTR of *CXXC*5 and LOC106505926, then regulate their expression.

### Biological characteristics of LOC106505926

**Fig. 4:**
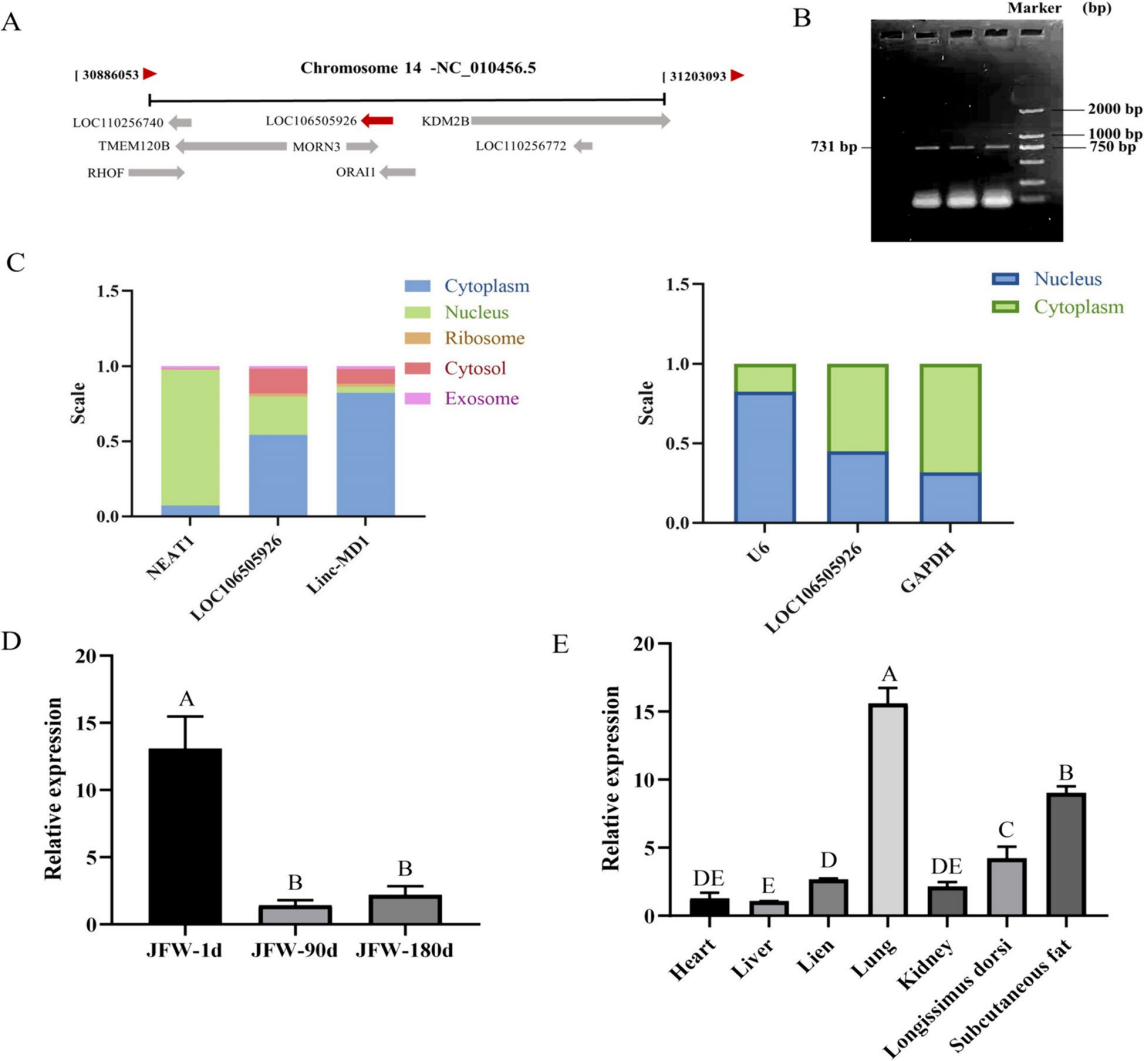
Biological characteristics of LOC106505926. **A** The location of LOC106505926 on the chromosome. **B** Agarose gel electrophoresis of LOC106505926. **C** The results of nuclear cytoplasmic localization of LOC106505926. **D** Temporal expression characteristics of LOC106505926. **E** The tissue expression characteristics of LOC106505926. Different uppercase indicates extremely significant differences. (Mean ± SD; Values with different letters indicated significant differences, image of gel was provided in the supplementary file 4)

LOC106505926 is situated on chromosome 14 of pigs (Fig. 4A). To confirm its presence, the cDNA from the longissimus dorsi muscle of Jinfen White pigs was used as a template to amplify LOC106505926, resulting in a 731 bp partial sequence (Fig. 4B). The subcellular localization of LOC106505926 was predicted using online software lncLocator, indicating that it predominantly resides in the cytoplasm. Subsequently, RNA was extracted from both the cytoplasm and nucleus of skeletal muscle satellite cells, revealing that LOC106505926 is mainly localized in the cytoplasm but also present in the nucleus (Fig. 4C). Analysis of the expression pattern of LOC106505926 showed a decrease in relative expression levels with increasing age in Jinfen White pigs (Fig. 4D). In addition, LOC106505926 has a high expression level in the lung, subcutaneous fat and longissimus dorsi muscle (Fig. 4E).

### LOC106505926 promotes the proliferation and differentiation of PSCs

**Fig. 5:**
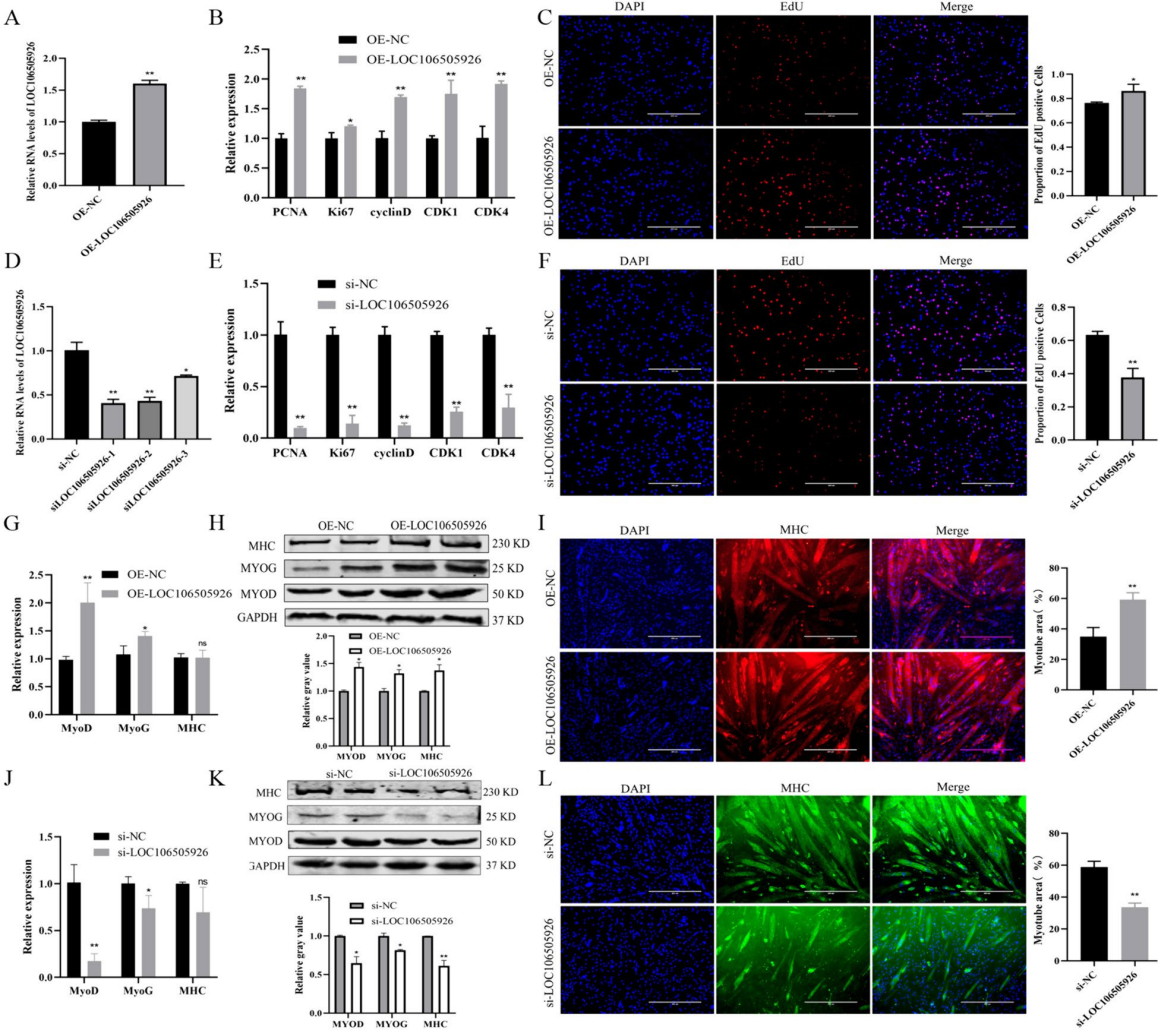
The effect of LOC106505926 on the proliferation and differentiation of PSCs. **A** Cell transfection efficiency of OE-LOC106505926. **B** The expression changes of proliferation marker genes after transfected with OE-LOC106505926. **C** The EdU results after transfected with OE-LOC106505926. **D** Cell transfection efficiency of siRNA of LOC106505926. **E** The expression changes of proliferation marker genes after transfected with si-LOC106505926. **F** The EdU results after transfected with si-LOC106505926. **G** The expression changes of differentiation marker genes after transfected with OE-LOC106505926 at mRNA level. **H** The expression changes of differentiation marker genes after transfected with OE-LOC106505926 at protein level. **I** The results of immunofluorescence after transfected with OE-LOC106505926. **J** The expression changes of differentiation marker genes after transfected with si-LOC106505926 at mRNA level. **K** The expression changes of differentiation marker genes after transfected with si-LOC106505926 at protein level. **L** The results of immunofluorescence after transfected with si-LOC106505926. (Mean ± SD; *P < 0.05, **P < 0.01. The blots were cut prior to hybridisation with antibodies, and images of blots for all replicates were provided in the supplementary file 4)

The overexpression vector of LOC106505926 was constructed and transfected into PSCs. The expression of LOC106505926 was significantly increased in OE-LOC106505926 group (Fig. 5A, *P* < 0.01). The expression of *PCNA*, *cyclin D*, *CDK*1 and *CDK*4 was significantly increased (Fig. 5B, *P* < 0.01), along with a significantly increase in the number of EdU-positive cells (Fig. 5C, *P* < 0.05). Three siRNA sequences of LOC106505926 were synthesized and detected the interference efficiency after transfection of PSCs (Fig. 5D). Among these sequences, siLOC106505926-1 was chosen for transfection into PSCs to evaluate the expression of proliferation marker genes. Compared with the si-NC group, the expression of proliferation marker genes was significantly decreased (Fig. 5E, *P* < 0.01), and the EdU assay revealed a significantly reduction in the number of positive cells (Fig. 5F, *P* < 0.01).

Then, the impact of LOC106505926 on PSC differentiation was investigated. The results showed that after transfected with OE-LOC106505926, there was a significant increase in *MyoD* expression (*P* < 0.01) and *MyoG* expression (*P* < 0.05), with no significant change in *MHC* expression (Fig. 5G). Western blot revealed OE-LOC106505926 transfection group had higher content of MYOD, MYOG, and MHC (Fig. 5H, *P* < 0.01). Immunofluorescence staining results showed the number of myotubes was significantly increased in the OE-LOC106505926 group (Fig. 5I, *P* < 0.01). Conversely, transfection with si-LOC106505926 yielded opposite results (Fig. 5J-L).

### *CXXC*5 promotes the proliferation and differentiation of PSCs

**Fig. 6:**
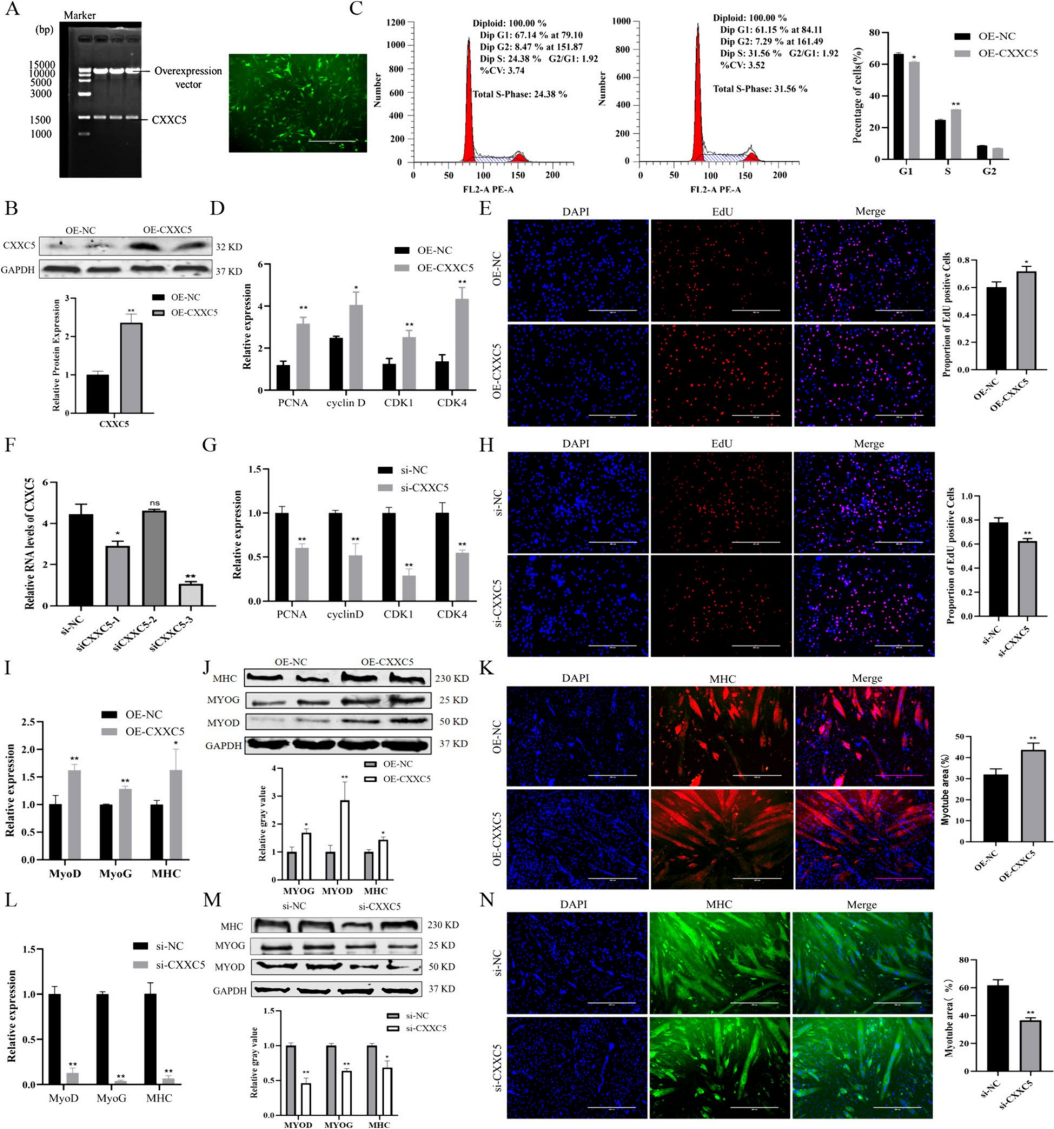
The effect of *CXXC*5 on the proliferation and differentiation of PSCs. **A** Construction of OE-CXXC5 overexpression vector and detection of transfection efficiency. **B** Transfection efficiency of OE-CXXC5 was detected by western blot. **C** Effect of OE-CXXC5 on cell cycle by flow cytometry. **D** The expression changes of proliferation marker genes after transfected with OE-CXXC5. **E** The results of EdU after transfected with OE-CXXC5. **F** Transfection efficiencies of siCXXC5-1, 2, 3 were detected by qRT-PCR. **G** The expression changes of proliferation marker genes after transfected with siCXXC5-3. **H** The results of EdU after transfected with siCXXC5-3. **I** The expression changes of differentiation marker genes after transfected with OE-CXXC5 at mRNA level. **J** The expression changes of differentiation marker genes after transfected with OE-CXXC5 at protein level. **K** The results of immunofluorescence after transfected with OE-CXXC5. **L** The expression changes of differentiation marker genes after transfected with siCXXC5 at mRNA level. **M** The expression changes of differentiation marker genes after transfected with siCXXC5 at protein level. **N** The results of immunofluorescence after transfected with siCXXC5. (Mean ± SD; *P < 0.05, **P < 0.01. The image of gel was provided in the Supplementary file 4, the blots were cut prior to hybridisation with antibodies, and images of blots for all replicates were provided in the Supplementary file 4)

The overexpression vector of *CXXC*5 was constructed and immunofluorescence results showed a positive outcome following transfection of PSCs (Fig. 6A). Compared with OE-NC group, the expression of CXXC5 protein in the OE-CXXC5 group was significantly enhanced (Fig. 6B, *P* < 0.01). The effect of *CXXC*5 on the cell cycle was detected by flow cytometry, the results showed that the proportion of S phase cells significantly increased (Fig. 6C, *P* < 0.01), accompanied with the significant upregulation of *PCNA*, *Cyclin D*, *CDK*1 and *CDK*4 (Fig. 6D, *P* < 0.05). EdU results showed that the number of positive cells significantly increased after transfection of OE-CXXC5 (Fig. 6E, *P* < 0.01). Three siRNA sequences of *CXXC*5 were designed for transfection into PSCs with siCXXC5-3 demonstrating the highest interference efficiency (Fig. 6F). Transfection of siCXXC5-3 in PSCs resulted in contrary outcomes compared to OE-CXXC5 transfection (Fig. 6G, H).

Transfected PSCs were differentiated in order to investigate the impact of *CXXC*5 on PSC differentiation. The expression levels of *MyoD*, *MyoG* and *MHC* were significantly increased after transfection with OE-CXXC5 (Fig. 6I, *P* < 0.05). This observation was further confirmed by Western blot (Fig. 6J, *P* < 0.05). The immunofluorescence assay showed a significantly increase in the number of myotubes in the OE-CXXC5 group compared with OE-NC group (Fig. 6K, *P* < 0.01). In addition, the effect of siCXXC5-3 on the differentiation of PSCs was investigated. The results were contrary to the transfection of OE-CXXC5 (Fig. 6L-N), suggesting that *CXXC*5 promotes the differentiation of PSCs.

### The rescue assay certified LOC106505926/miR-22-5p/*CXXC*5 regulates PSCs proliferation and differentiation

**Fig. 7:**
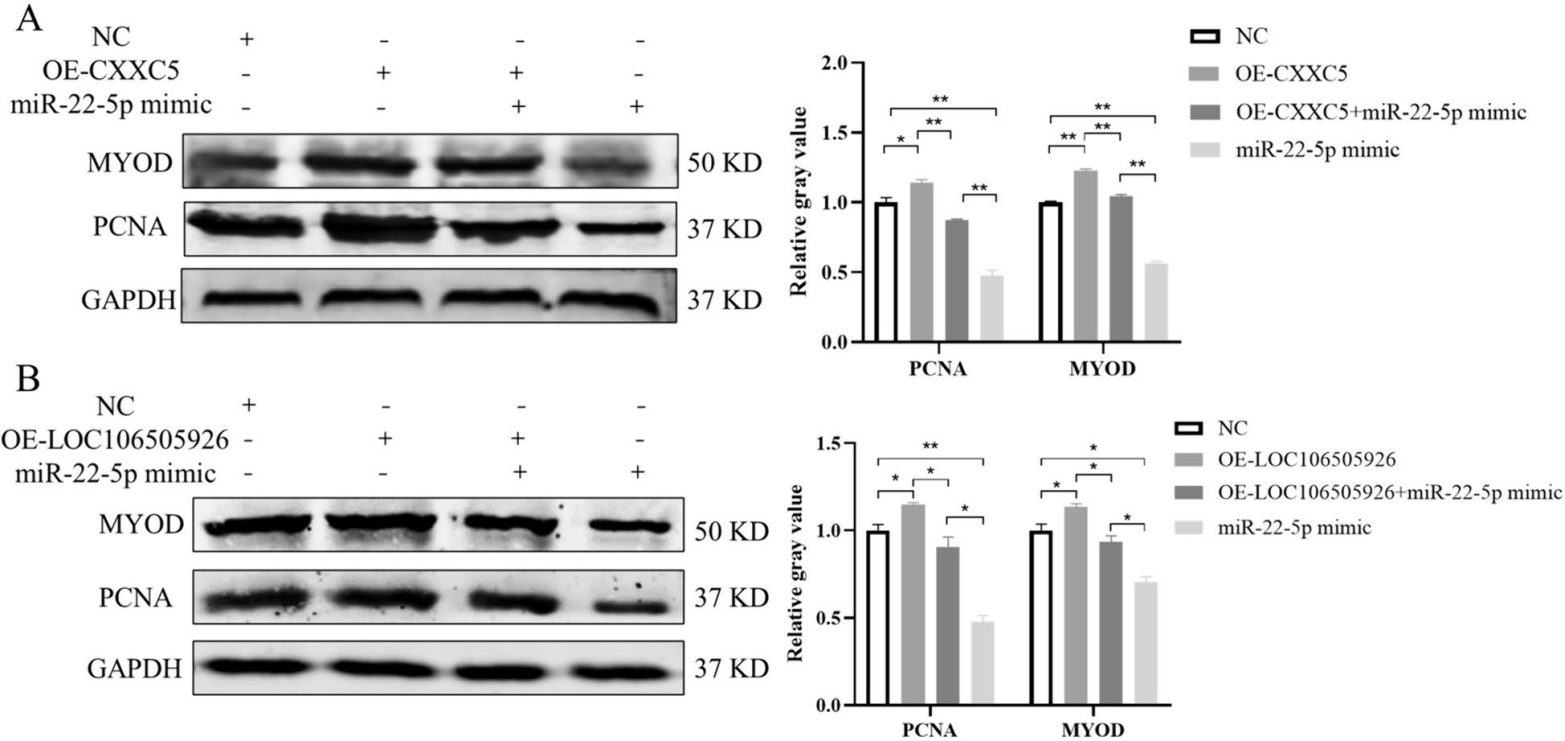
Changes of proliferation and differentiation marker genes in rescue experiment. **A** Detection of PCNA and MYOD expression after transfection of OE-CXXC5 and miR-22-5p at protein level. **B** Detection of PCNA and MYOD expression after transfection of OE-LOC106505926 and miR-22-5p at protein level. (Mean ± SD; *P < 0.05, **P < 0.01. The blots were cut prior to hybridisation with antibodies, and images of blots for all replicates were provided in the supplementary file 4)

To further explore the relationship between miR-22-5p and *CXXC*5, rescue experiments were performed. Overexpression of *CXXC*5 alleviates the inhibitory effect of miR-22-5p on the expression of PCNA and MYOD (Fig. 7A). In addition, transfected with OE-LOC106505926 also alleviates the inhibitory effect of miR-22-5p on the proliferation and myogenic differentiation of PSCs (Fig. 7B). Thus, LOC106505926 may reduce the inhibitory effect of miR-22-5p on *CXXC*5 by competitively binding miR-22-5p.

### LOC106505926 inhibits preadipocyte differentiation and directly interacts with FASN

**Fig. 8:**
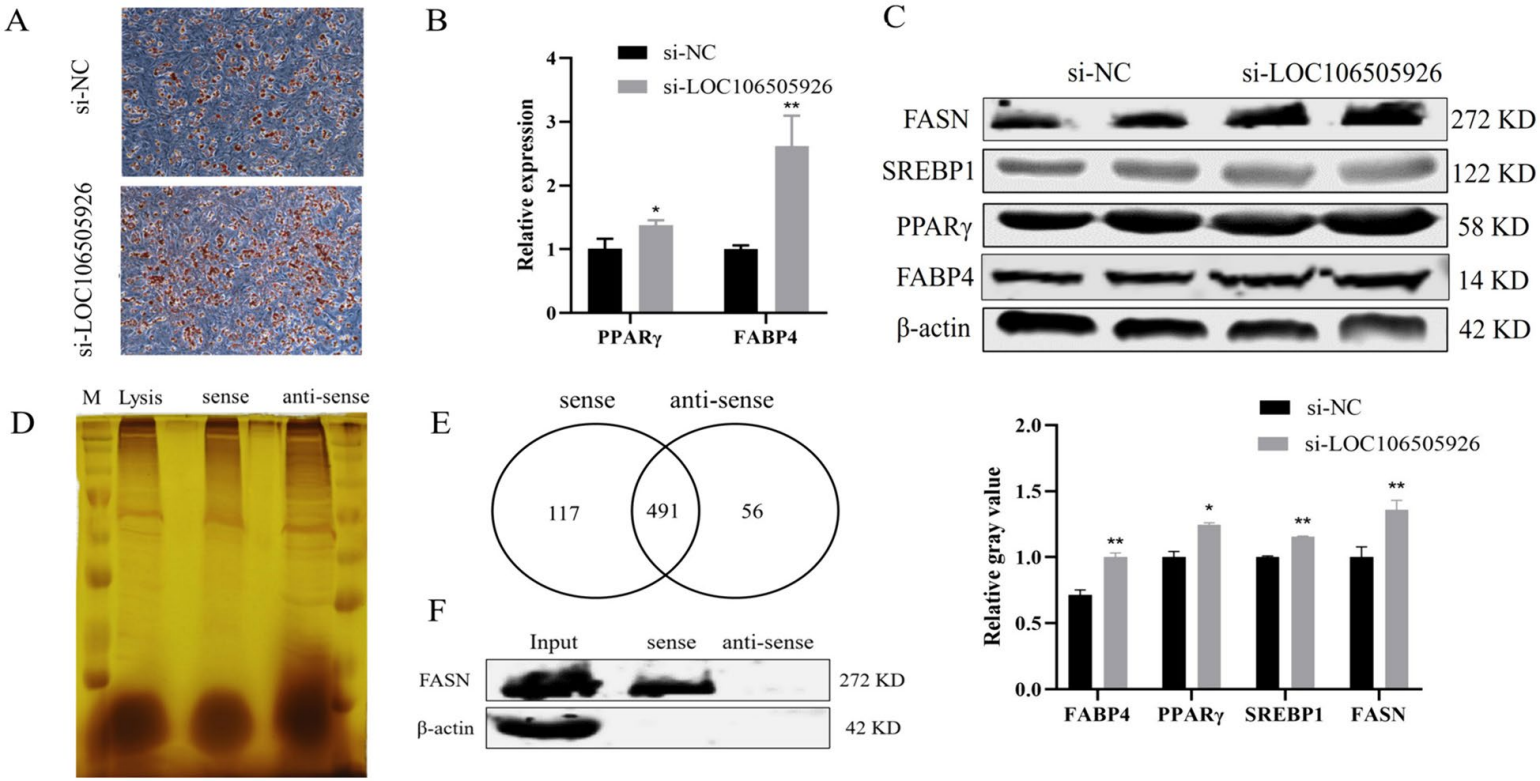
The effect of LOC106505926 on the differentiation of preadipocytes. **A** Cell oil red O staining. **B** The expression changes of differentiation marker genes after transfected with si-LOC106505926 at mRNA level. **C** The expression changes of differentiation marker genes after transfected with si-LOC106505926 at protein level. **D** Protein silver staining gel map. **E** Venn diagram of mass spectrometry detection of the pull-down protein. **F** LOC106505926 interacts with FASN. (Mean ± SD; *P < 0.05, **P < 0.01. The blots were cut prior to hybridisation with antibodies, and images of blots for all replicates were provided in the supplementary file 4)

After si-LOC106505926 was transfected in porcine precursor adipocytes, the obtained cells were subjected to lipogenic differentiation. Oil Red O staining showed that LOC106505926 could inhibit lipid-droplet formation (Fig. 8A), the mRNA and protein levels of lipogenic factors were dramatically elevated in the si-LOC106505926 group compared with the si-NC group (Fig. 8B, C). RNA pull-down assay was performed with biotinylated LOC106505926 to identify the LOC106505926 interacting proteins. After SDS-PAGE, the gel was stained with silver nitrate (Fig. 8D) and analyzed by MS (Fig. 8E). The proteins that specifically bind to LOC106505926 (including FASN) were identified. The association of LOC106505926 with FASN was validated by Western blot, and the results showed that the target protein FASN was detected in LOC106505926 pull-down protein samples but not in the samples associated with antisense LOC106505926 (Fig. 8F).

## Discussion

In recent years, there were many examples that miRNAs affect skeletal muscle development by participating in various processes. For example, miR-142a-5p was an important regulator of denervation-induced skeletal muscle atrophy via targeting MFN1 [28]. MiR-135a-5p regulated skeletal muscle fibrosis by Tgfbr2/Smad4 signaling pathway [29]. MiR-491 can specifically bind to the 3′UTR of myomaker and regulates myocyte differentiation and muscle regeneration [30]. Previous studies demonstrated that the expression of miR-22-5p in mice was significantly up-regulated with the increase of age [31]. MiR-22-5p has also been testified to play important roles in a variety of diseases, such as acute myocardial infarction, myocardial damage and hepatocellular carcinoma [32–34]. MiR-22-5p regulated the proliferation of human osteoblasts via MALAT1 and VEGF [35]. Many miRNAs have been proven to be involved in the regulation of myoblast proliferation and differentiation [36–39]. At present, the regulation of myogenesis by miR-22-5p remains unclear. In this study, we demonstrated that miR-22-5p was differentially expressed in the LDM of Jinfen White pigs at three developmental stages, suggesting its involvement in the regulation of porcine muscle development.

*CXXC*5 is a member of the CXXC-type zinc finger protein family, which has a CXXC-type zinc finger (ZF-CxxC) protein structural domain. The CXXC structural domain normally binds to unmethylated CpG islands in gene promoters and plays a key role in epigenetic regulation [40, 41]. *CXXC*5 does not have any catalytic domain, but it has been shown to regulate transcription by directly binding to DNA [42]. *CXXC*5 was expressed in a variety of tissues [43], and was involved in the development of nephron and heart, as well as the differentiation of osteoblasts, oligodendrocytes and endothelial cells [44–46]. Recent studies showed that *CXXC*5 affected cell cycle regulation and was required for myeloid cell maturation and differentiation [47]. In addition, *CXXC*5 regulates multiple physiological processes via several important cellular signaling pathways. It was demonstrated that* CXXC*5 mediated the BMP signaling pathway and induced endothelial cell differentiation and vessel formation [45]. *CXXC*5 also participated in the Wnt signaling pathway to regulate the differentiation of neural stem cells [48] and osteoblast [44]. Moreover, the ZF-CXXC domain of *CXXC*5 interacted with the SMAD2/3/4 and activated TGF-β signaling and BMP signaling to promote normal heart development [49] and mediate TNF-α induced apoptosis [50]. Besides, *CXXC*5 was involved in cell cycle arrest and DNA repair by activating ATM-p53 signaling axis [51]. Therefore, *CXXC*5 may be an important medium for various signal inputs.

Interestingly, *CXXC*5 played a crucial role in the myogenic differentiation of C2C12 cells [52]. Previous research has highlighted the involvement of* CXXC*5 in regulating skeletal muscle regeneration through the TGF-β, Wnt, Vitamin D signaling pathways [50, 53–55]. Wu’s study further suggested a potential link between *CXXC*5 and skeletal muscle development, as well as Ca2 + release in muscle, using whole-genome sequencing in Qingyu pigs [23]. Our current findings support previous evidence by demonstrating that *CXXC*5 promotes the proliferation and differentiation of PSCs. Moreover, our study revealed that miR-22-5p inhibits the proliferation and differentiation of PSCs by binding to the 3′UTR of *CXXC*5.

With the discovery of a large number of lncRNAs, people have gradually realized that lncRNA has a very important regulatory function and can participate in various biological processes and signaling pathways. In recent years, research has showed that lncRNA played an important role in transcription as a transcriptional regulator [56], regulating chromatin stability [57], the development of subcellular organs [58] and miRNA expression [59]. Studies have found that lncRNA-TBP promotes myoblast differentiation, reduces fat deposition, activates slow muscle phenotype, and induces muscle hypertrophy. It is worth noting that lncRNA-TBP, as a regulatory RNA, directly interacts with TBP protein to regulate TBP-target genes [60]. In a study of lipogenic transdifferentiation of myoblasts, 114 core lncRNAs were identified, lncRNA-GM43652 gene was a potential regulator of adipogenesis in muscle cells [61]. In our study, LOC106505926 regulates the differentiation of satellite cells through the ceRNA mechanism, while in adipocytes, it inhibits the differentiation of preadipocytes by directly binding to FASN. Our study provides evidence for a variety of biological functions and specific mechanisms of lncRNAs.

## Conclusion

The present study demonstrated that miR-22-5p inhibited the proliferation and differentiation of PSCs via binding to the 3′UTR of *CXXC*5. LOC106505926 was found to play a role in the proliferation and differentiation of PSCs as a molecular sponge of miR-22-5p. In addition, LOC106505926 inhibited the differentiation of preadipocytes by directly binding to FASN. These results provide insights into the molecular regulation mechanism in porcine skeletal muscle development and fat deposition.

## Materials and methods

### Experimental animals and samples

All experimental protocols were approved by the Ethics Committee of Shanxi Agricultural University (Shanxi, China, Approval No. SXAU-EAW-2021Sus.AB.0926001). A total of nine healthy Jinfen White pigs (castrated boars) at 1, 90, and 180 days of age (3 pigs per age) used in the experiment came from Datong Pig Breeding Farm (Shanxi, China) and were raised under standard conditions without restriction on feeding and drinking. These pigs were named JFW_1d_1, JFW_1d_2, JFW_1d_3; JFW_90d_1, JFW_90d_2, JFW_90d_3; JFW_180d_1, JFW_180d_2, JFW_180d_3, respectively and sacrificed by electric shock and neck exsanguination on days 1, 90 and 180, respectively. Seven tissues including heart, liver, spleen, lung, kidney, longissimus dorsi, and subcutaneous fat were collected strictly according to the anatomical structure of the pigs and instantly frozen in liquid nitrogen. All samples were stored at -80 ℃ for later use.

### Construction of cDNA library and sequencing

The LDM samples of Jinfen White pigs at 1,90 and 180 days (*n* = 3) were used to construct a sequencing library. Total RNA was extracted from LDM tissues using RNAiso Plus following the recommended protocol. Following quality evaluation, mRNA was isolated from the total RNA and fragmented. Subsequently, first strand cDNA was synthesized using random hexamer primer and M-MuLV Reverse Transcriptase (RNase H). Second strand cDNA synthesis was subsequently carried out using DNA Polymerase I and RNase H. After adenylation of the 3’ end of the DNA fragment, the NEBNext Adaptor was ligated for hybridization preparation. Library quality was assessed using a Bioanalyzer 2100 system, sequencing was performed on the Illumina Hiseq platform and 125 bp/150 bp paired-end reads were generated. The sequencing data is publicly available in the NCBI SRA database (https://www.ncbi.nlm.nih.gov/sra/), with accession number: PRJNA867525.

### Construction of small RNA library and sequencing

For small RNA sequencing, the 3’ SR adaptor was directly ligated to 3’ end of small RNA and 5’ ends adapter was ligated to 5’ ends of small RNA, then, first strand cDNA was synthesized. The DNA fragments of 140–160 bp were recovered, and the library quality was evaluated using a DNA High Sensitivity Chip on the Agilent Bioanalyzer 2100 system. The sequencing was performed on the Illumina Hiseq platform and 50 bp single-end reads were gained. The sequencing data is also publicly available in the NCBI SRA database (https://www.ncbi.nlm.nih.gov/sra/), with accession number: PRJNA867525.

### Differentially expressed RNA identification and pathway analysis

HISAT2 (version 2.0.2) was used to get the clean reads mapped to the pig reference genome (Sus scrofa 11.1). The mRNAs and lncRNAs were normalized according to their fragments per kilobase of exons per million mapped reads (FPKM). The expression level of miRNAs was calculated using the HTseq software (0.6.1), transcript per million (TPM) values were used to determine miRNA counts and levels of expression [62]. Subsequently, differentially expressed mRNAs, differentially expressed lncRNA and differentially expressed miRNA were identified using the R packages DEG seq2 [63].

To explore the function of the miRNAs, potential target genes of miRNAs were identified. Then, the Gene Ontology (GO) terms and Kyoto Encyclopedia of Genes and Genomes (KEGG) were performed using the DAVID tool, *P* < 0.05 were considered significant.

### Construction of lncRNA-miRNA-mRNA networks

The miRanda software (v3.3a) was used for predicting the target genes and lncRNAs for miRNAs. Then, the correlation between gene nodes was calculated by Pearson correlation coefficient. Cytoscape (v3.5.1) visualized the nodes of the network. RNAhybrid (https://bibiserv.cebitec.uni-bielefeld.de/rnahybrid) was used to predict the binding site secondary structure between miRNAs and its targets.

### PSCs isolation, culture and differentiation

PSCs were isolated from 3-day-old Jinfen White male pigs. The skin of pigs was disinfected with 75% alcohol after slaughter. The LDM were collected and kept in PBS supplement with 1% penicillin-streptomycin. The tissue was digested with digestive solution (DMEM containing 15% collagenase II, 15% dispase II, 1% penicillin-streptomycin) at 37 ℃ for 1 h, and the digestion was terminated with DMEM (including 10% FBS). The mixture was filtered through a 100 μm cell strainer and centrifuged at 1500 r/min for 10 min. Then, the supernatant was removed and the precipitate was resuspended with PBS. Next, 70 and 40 μm cell strainers were used to filter the suspension. The supernatant was removed and resuspended with erythrocyte lysate, an equal volume of PBS was added, centrifuged at 1000 r/min for 5 min. Last, the cells were suspended with 20% FBS (Gibco, Life Technologies, United States) and 1% penicillin-streptomycin. Cells were cultured in thermostatic incubator (Thermo Fisher, United States) for 1 h, the cell suspension was transferred to the new culture dishes, which were coated with Matrigel (Corning, United States).

Isolated PSCs were cultured on Matrigel coated 10 cm plates (Corning, United States) with the medium (containing 20% FBS, 1% penicillin-streptomycin, 2.5 ng/mL human recombinant basic fibroblast growth factor) (Gibco, United States). When the cells density was up to 70%, the differentiation medium (containing 5% horse serum) (HyClone, United States) was used to induce cell myogenic differentiation, the medium was changed every 2 days.

### Porcine preadipocytes isolation, culture and differentiation

The subcutaneous adipose tissue of piglets was collected and placed in a sterile petri dish to remove fascia and connective tissue. Then, the tissue was cut into pieces and digested with 2 mg/mL type I collagenase (Gibco, USA) for 1 h, the digestion was terminated with DMEM (including 10% FBS). After filtration, the cells were centrifuged at 1000 r/min for 10 min, resuspended and inoculated into a petri dish for culture. Preadipocytes were cultured with DMEM (including 10% FBS, 1% penicillin-streptomycin). When the cell density reached 80 − 90%, the preadipocytes were digested with 0.25% trypsin. Replace the medium every two days. When the cell density reached 95%, lipogenic induction was performed. The complete induction medium was supplemented with dexamethasone (1 µmol/L), indomethacin (100 µmol/L), IBMX (0.5 mmol/L) and insulin (10 µg/mL). After 4 days of differentiation, the maintenance medium was replaced. The maintenance medium was a complete medium supplemented with insulin (10 µg/mL). After that, the maintenance medium was replaced every 2 days, and the cells were observed under a microscope.

### Nuclear and cytoplasmic RNA fractionation

PSCs were collected and washed twice with cold PBS and centrifuged at 500 g for 3 min. The precipitate was resuspended with 0.3 mL Cell Fractionation Buffer, placed on ice for 10 min, and centrifuged at 500 g at 4 °C for 3 min. The supernatant was transferred to a new 1.5 mL micro-centrifugal tube without RNase, and 0.3 mL 2×Lysis/Binding Buffer was added and mixed, which was used as cytoplasmic RNA. The precipitate was washed with 0.3 mL Cell Disruption Buffer, and 0.3 mL 2×Lysis/Binding Buffer was added after shaking and mixing to obtain nuclear RNA. Then, 0.3 mL absolute ethanol was added to the cytoplasm and nucleus RNA, poured into the filter extraction, centrifuged at 1,400 r/min for 1 min, discarded the liquid, added Wash Solution centrifuged at 1,400 r/min for 1 min, repeated several times, 0.3 mL Elution Solution was poured into the filter, replaced the new centrifuge tube, centrifuged at 10,000 r/min for 30 s to obtain RNA and stored at -80 °C.

### Cell transfection

Firstly, the overexpression vector of *CXXC*5: pHBLV-CXXC5-puro (OE-CXXC5) and control vector of *CXXC*5: pHBLV-CMVIE-puro control (NC-CXXC5) were constructed and synthesized. When the density of HEK-293T cells reached to 70% confluence, the Lipo3000 (Invitrigen, USA) was used to transfect the plasmid and two package plasmids psPAX2 and pMD2G (Public Protein/Plasmid Library, Jiangsu, China) at the ratio of 1:1:1 for 6 h following the introduction. The supernatants were collected and stored at -80 ℃ for infection. After 48 h of transfection, the fluorescence rate was observed. The overexpression vector of LOC106505926 was synthesized by Tsingke Biotechnology according to the sequence on NCBI. The RNA oligo against of porcine *CXXC*5, miR-22-5p and LOC106505926 were purchased from GenePharma (Shanghai, China). The 50 nM RNA oligo specific were transfected to cells at a density about 60%. The siRNA sequences were shown in the supplementary Table S2.

### Total RNA extraction and quantitative real-time polymerase chain reaction (qRT-PCR)

Total RNA was extracted from samples according to the instruction of TaKaRa RNAiso Plus (Takara, Japan). The cDNA was synthesized using PrimeScript RT reagent Kit with gDNA Eraser (Takara, Japan). The relative expression level of genes was normalized by *18sRNA*. Primers′ information was shown in supplementary Table S3. The qRT-PCR reaction system: cDNA 2 µL, 2×SYBR Premix Ex Taq II 10 µL, forward and reverse primers 0.5 µL, RNAase Free ddH_2_O supplemented to 20 µL. Reaction procedure: 95 °C 30 s; 95 °C 5 s, 58 °C 30 s, 35 cycles; the melting curve program is 95 °C 15 s, 60 °C 35 s, 95 °C. Each sample was repeated three times. For miRNAs, U6 snRNA was selected as the internal control. The primers of miRNAs were designed using the stem-loop approach and the information of primers was shown in supplementary Table S3. The cDNA was synthesized according to the protocol of miRNA 1st Strand cDNA Synthesis Kit (Vazyme, China). Each 20 µL qRT-PCR reaction mixture contained 2×miRNA Universal SYBR qPCR Master Mix 10 µL (Vazyme, China, MQ101), cDNA 1 µL, nuclease-free H_2_O 7.8 µL, 0.5 µL specific primer and mQ primer R. The following parameters were used for qRT-PCR: pre-denaturation for 5 min at 95 ℃, then 40 cycles of 95 ℃ for 10 s, 60 ℃ for 30 s, and 72 ℃ for 8 s, the melting curve program is 95 °C 15 s, 60 °C 60 s, 95 °C 15 s. Relative expression levels of genes and miRNAs were calculated by the 2^−ΔΔCt^ method.

### Flow cytometry

After 48 h of transfection, the cells were collected and resuspended with 75% ethanol when the cell density reached 90%. The cell suspension was placed at -20 °C for 10 h. After centrifugation, RNase A was added and incubated at 37 °C for 30 min. PI staining solution was added and incubated at 4 °C for 30 min. The proportion of cells in different cell cycles was analyzed by flow cytometry.

### EdU assay

PSCs were incubated with EdU-containing medium for 2 h, then, fixed with 4% paraformaldehyde for 30 min, washed with PBS, incubated with 2 mg/mL glycine for 5 min, washed with PBS for 5 min, incubated with 0.5% TritonX-100 for 10 min, then incubated with Apollo staining reaction solution (Ribobio, China) for 30 min, washed with PBS and stained with DAPI reaction solution for 10 min. Observed with a fluorescence microscope.

### Dual-luciferase reporter assay

The wild-type of 3′UTR of CXXC5 and mutant-type of 3′UTR of CXXC5 were constructed and inserted into psi-CHECK2 vectors, then, named “CXXC5-wt-psiCHECK2” and “CXXC5-mut-psiCHECK2”. HEK-293T cells were seeded and cultured on 24-well plates. Then, cells were co-transfected with the miRNAs (mimic NC, miR-22-5p mimic, inhibitor NC or miR-22-5p inhibitor) and the CXXC5-wt-psiCHECK2 or CXXC5-mut-psiCHECK2 vectors. The cells were collected after 24 h of cell transfection. A standard plate reader (BioTek, Vermont, United States) was used to measure the luciferase activity.

### Western blot

In PSCs, the expression levels of MYOG, MYOD, MHC and CXXC5 protein were detected. The protein expression levels of FABP4, PPARγ, SREBP1 and FASN were detected in preadipocytes. Transfected cells were lysed in RIPA buffer with 1% PMSF. 5×Buffer was added to the sample and denatured at 100 °C for 10 min. SDS-PAGE gel electrophoresis was performed at 80 V 30 min, 120 V 90 min. Then transferred them onto a PVDF membrane and non-specific binding was blocked with 5% non-fat milk in PBS for 1 h. Then, they were incubated with 1:1000 diluted polyclonal rabbit MYOG (Abclonal, China), MYOD (Proteintech, China), CXXC5 (Bioss, China), 1:500 diluted polyclonal mouse MHC (DSHB, America), 1:1000 diluted polyclonal rabbit FABP4 (Proteintech, China), PPARγ (Proteintech, China), SREBP1 (Proteintech, China) and FASN (Proteintech, China) at 4 ℃ overnight. The blots were subsequently incubated with secondary antibody (1:10000) for 1 h. Secondary antibody include goat anti-mouse IgG (Servicebio, China) and goat anti-rabbit IgG (Servicebio, China). GAPDH (Servicebio, China) was used as an endogenous protein for normalization. Image J software was used to conduct quantitative analysis of western blot results according to the gray value of the strip.

### Immunofluorescent analysis

Cells were fixed with precooled 4% paraformaldehyde for 30 min. Then permeabilized in 0.5% Triton X-100 for 10 min. After that, cells were blocked with 3% bovine serum albumin (BSA), and incubated with 1:1000 diluted polyclonal mouse MHC (DSHB, America) antibodies overnight at 4 ℃. Cells were washed with PBS for 1–3 times and incubated with goat anti-mouse IgG antibodies (Bioss, China) for 1 h at room temperature. Then, the cells were stained with Hoechst 33,342 (Sanofi-Aventis, Germany) for 10 min. Images were acquired by a Leica SP8 confocal microscope. Immunofluorescence results were quantified by Image J software.

### Oil red O staining

After lipogenic differentiation of porcine preadipocytes, oil red O staining was performed, 4% paraformaldehyde was added, fixed at room temperature for 30 min, and washed with PBS. Oil red O dye solution (Solarbio, China) was added and incubated at room temperature for 30 min. After washing with PBS, the cells were observed under an inverted microscope. Isopropanol was added to extract triglyceride, and the OD value was detected by enzyme-labeled instrument.

### Silver nitrate staining

Take out the gel after SDS-PAGE, put it in the staining box, wash the gel twice with distilled water, then pour out the distilled water, add 25% ethanol to denature for 3 min, recover the ethanol, wash the gel with distilled water, add 0.1% silver nitrate solution, incubate for 30 min on the shaker, and fix the gel on the glass plate to observe the staining.

### RNA pull-down assay

The biotinylated probe of LOC106505926 was synthesized by RiboBio. Preparation of cell lysates with standard lysis buffers, wash the beads with 20mM Tris (pH 7.5), add an equal volume of 1×RNA Capture Buffer, resuspend beads by pipetting or vortexing, then, add 50 pmol of labeled RNA to the beads, mix gently by pipetting, incubate for 15–30 min at room temperature with agitation. The RNAs were targeted with streptavidin beads, add 100 µL of Master Mix (including Protein-RNA Binding Buffer, glycerol, salts, lysate, nuclease-free water) to the RNA-bound beads, incubate 30–60 min at 4 °C with agitation or rotation. The protein complexes were obtained after several elutions. Finally, the protein complexes associated with the beads were analyzed by mass spectrometry (MS) and western blot.

### Statistical analysis

All results were presented as mean ± standard error of mean (SEM). Multigroup comparisons of the means were carried out by a one-way analysis of variance test. The two-tailed t-test was performed for differences analysis between the two groups. * represents *P* < 0.05, ** represents *P* < 0.01.

### Supplementary Information

Supplementary Material 1.

Supplementary Material 2.

Supplementary Material 3.

Supplementary Material 4.

## Data Availability

Sequence data that support the findings of this study have been deposited in SRA Database (BioProject ID: PRJNA867525).

## Abbreviations

LDM: Longissimus dorsi muscle
PSCs: Porcine skeletal muscle satellite cells
LncRNA: Long non-coding RNA
GO: Gene ontology
KEGG: Kyoto encyclopedia of genes and genomes
CXXC5: CXXC finger protein 5
MRFs: Myogenic regulatory factors
MYOD: Myogenic differentiation
MYOG: Myogenin
MHC: Myosin heavy chain
MEF2: Myocyte enhancer factor 2
Myf5: Myogenic factor 5
Myf6: Myogenic factor 6
GAPDH: Glyceraldehyde-3-phosphate dehydrogenase
PPARγ: Peroxisome proliferator-activated receptorγ
FABP4: Fatty acid binding protein 4
SREBP1: Sterol regulatory element binding transcription factor 1
FASN: Fatty acid synthase
