# Supplementary material for: Effect of LncRNA LOC106505926 on myogenesis and Lipogenesis of porcine primary cells
Source: BMC Genomics. 2024 May 30;25:530. doi: 10.1186/s12864-024-10422-y (PMC11137989; doi:10.1186/s12864-024-10422-y)
Supplement: Supplementary file 1 — Supplementary Material 1. [file 12864_2024_10422_MOESM1_ESM.docx]

Table S1 Quality assessment of sRNA sequencing data

| Sample name | Total reads | low quality | 5_adapter_co-ntamine | | | 3_adapter null or insert null | with ploy A/T/G/C | clean reads |
| --- | --- | --- | --- | --- | --- | --- | --- | --- |
| JFW-1 d-1 | 14502686 | 31195 | | 2890 | | 216516 | 4054 | 14248031 |
| JFW-1 d-2 | 12895691 | 30560 | 1547 | | 485250 | | 2038 | 12376295 |
| JFW-1 d-3 | 15956038 | 55226 | 1751 | | 151610 | | 3878 | 15743573 |
| JFW-90 d-1 | 18990473 | 52479 | 2540 | | 168886 | | 8732 | 18757835 |
| JFW-90 d-2 | 17604307 | 57598 | 3911 | | 193692 | | 4551 | 17344555 |
| JFW-90 d-3 | 16432480 | 50812 | 4385 | | 156206 | | 11274 | 16209802 |
| JFW-180 d-1 | 16304830 | 51453 | 2182 | | 89966 | | 7347 | 16153882 |
| JFW-180 d-2 | 15114378 | 86154 | 1599 | | 200726 | | 7146 | 14818753 |
| JFW-180 d-3 | 13803894 | 46314 | 1992 | | 111389 | | 4124 | 13638295 |

Continued Table S1 Quality assessment of sRNA sequencing data

| Sample name | Reads | Bases | Error rate | Q20 | Q30 | GC content |
| --- | --- | --- | --- | --- | --- | --- |
| JFW_1d_1 | 14502686 | 0.725G | 0.01% | 99.09% | 97.26% | 50.78% |
| JFW_1d_2 | 12895691 | 0.645G | 0.01% | 99.14% | 97.47% | 49.64% |
| JFW_1d_3 | 15956038 | 0.798G | 0.01% | 99.13% | 97.26% | 49.71% |
| JFW_90d_1 | 18990473 | 0.950G | 0.01% | 99.10% | 97.29% | 47.46% |
| JFW_90d_2 | 17604307 | 0.880G | 0.01% | 99.11% | 97.24% | 47.86% |
| JFW_90d_3 | 16432480 | 0.822G | 0.01% | 99.11% | 97.30% | 47.37% |
| JFW_180d_1 | 16304830 | 0.815G | 0.01% | 99.15% | 97.26% | 48.07% |
| JFW_180d_2 | 15114378 | 0.756G | 0.01% | 97.86% | 93.96% | 48.47% |
| JFW_180d_3 | 13803894 | 0.690G | 0.01% | 98.91% | 96.38% | 48.13% |
