# Supplementary material for: Effect of LncRNA LOC106505926 on myogenesis and Lipogenesis of porcine primary cells
Source: BMC Genomics. 2024 May 30;25:530. doi: 10.1186/s12864-024-10422-y (PMC11137989; doi:10.1186/s12864-024-10422-y)
Supplement: Supplementary file 2 — Supplementary Material 2. [file 12864_2024_10422_MOESM2_ESM.docx]

Table S2. The siRNA sequences.

| Name | Sequence |
| --- | --- |
| siCXXC5-1 | 5′-CCCUACCUCAAAGCACAAATT-3′  5′-UUUGUGCUUUGAGGUAGGGTT-3′ |
| siCXXC5-2 | 5′-GGCCAUCAGAUUUGCAAAUTT-3′  5′-AUUUGCAAAUCUGAUGGCCTT-3′ |
| siCXXC5-3 | 5′-GAGGUAACGACGAAUCUAUTT-3′  5′-AUAGAUUCGUCGUUACCUCTT-3′ |
| si-LOC106505926-1 | 5′-GCCUUUCUUUGUCCUUUGUTT-3′  5′-ACAAAGGACAAAGAAAGGCTT-3′ |
| si-LOC106505926-2 | 5′-CAGAGAGGGUAAGUAAUUATT-3′  5′-UAAUUACUUACCCUCUCUGTT-3′ |
| si-LOC106505926-3 | 5′-GAGGUAACGACGAAUCUAUTT-3′  5′-AUAGAUUCGUCGUUACCUCTT-3′ |
| Negative control (NC) siRNA | 5′-UUCUCCGAACGUGUCACGUTT-3′  5′-ACGUGACACGUUCGGAGAATT-3′ |
| miR-22-5p mimics | 5′­-AGUUCUUCAGUGGCAAGCUUUA-3′  5′-AAGCUUGCCACUGAAGAACUUU-3′ |
| miR-22-5p inhibitor | 5′-UAAAGCUUGCCACUGAAGAACU-3′ |
| mimics NC | 5′­-UUCUCCGAACGUGUCACGUTT-3′  5′-ACGUGACACGUUCGGAGAATT-3′ |
| inhibitor NC | 5′-CAGUACUUUUGUGUAGUACAA-3′ |
