# Supplementary material for: Effect of LncRNA LOC106505926 on myogenesis and Lipogenesis of porcine primary cells
Source: BMC Genomics. 2024 May 30;25:530. doi: 10.1186/s12864-024-10422-y (PMC11137989; doi:10.1186/s12864-024-10422-y)
Supplement: Supplementary file 3 — Supplementary Material 3. [file 12864_2024_10422_MOESM3_ESM.docx]

Table S3 Primers of RT-qPCR

| Genes | Primer sequence (5′ -3′) | | Prouduct Length |
| --- | --- | --- | --- |
| CXXC5 | | F: AGAAGGTGATGCTTCCGACG | 137 bp |
|  |  | R: CATCCGTTTTGTCTTCGCCC |  |
| ssc-miR-22-5p | | Loop: GTCGTATCCAGTGCAGGGTCCGA  GGTATTCGCACTGGATACGACTAAAGC | 72 bp |
|  |  | F: CGCGAGTTCTTCAGTGGCAA |  |
|  |  | R: AGTGCAGGGTCCGAGGTATT |  |
| *PCNA* | | F: ATGCAGACACCTTGGCACTA | 155 bp |
|  |  | R: ACGTGCAAATTCACCAGAAGG |  |
| *Cyclin D* | | F: GGCGTGTGACCTTCACCTTA | 88 bp |
|  |  | R: AGCCTCTTTCAGCATCGGAC |  |
| *CDK*1 | | F: TGGGAGCTTAAGGAGAGCGAC | 88 bp |
|  |  | R: AGTGGATGTGGTAGATCCCAG |  |
| *CDK*4 | | F: GGCCCTCAAGAGCGTAAGAG | 165 bp |
|  |  | R: GTCTCTCGATCAGTTCGGGC |  |
| *MyoD* | | F: GGGTCGGATCCGTCTGAGC | 67 bp |
|  |  | R: CAGTGCGTGTCGTGGAGT |  |
| *MyoG* | | F: GAGCTGTATGAGACATCCCCC | 75 bp |
|  |  | R: GTGGACGGGCAGGTAGTTTT |  |
| *MHC* | | F: AAGGCATCATCAAGGACACTC | 106 bp |
|  |  | R: TGCGGCAGGTTGGCTCT |  |
| 18s RNA | | F: ATGCCAGAGTCTCGTTCGTTAT | 119 bp |
|  |  | R: CGGACAGGATTGACAGATTGAT |  |
| U6 | | F: CTCGCTTCGGCAGCACA | 60 bp |
|  |  | R: CTCGCTTCGGCAGCACA |  |

Note: Loop is a miRNA stem-loop reverse transcription primer.
