# Supplementary material for: Effect of LncRNA LOC106505926 on myogenesis and Lipogenesis of porcine primary cells
Source: BMC Genomics. 2024 May 30;25:530. doi: 10.1186/s12864-024-10422-y (PMC11137989; doi:10.1186/s12864-024-10422-y)
Supplement: Supplementary file 4 — Supplementary Material 4. [file 12864_2024_10422_MOESM4_ESM.docx]

**The original images of western blot and gels**

1. Prestained Protein Marker II (G2058, 10-180 kDa) that we used was shown below:


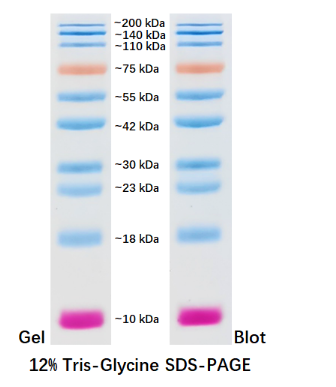


2. The original gels of the western blots in manuscript. (M: protein marker L: protein lanes)

**Figure 2H:** L1, L2: mimic NC; L3, L4: miR-22-5p mimic

**Figure 2K:** L1, L2: Inhibitor NC; L3, L4: miR-22-5p inhibitor

**Figure 4B:** The DNA Maker was shown on the left and the gel was shown on the right. L1, L2, L3:LOC106505926 (M: DNA marker L: DNA lanes)

**M**

**L2**

**L3**

**L1**

**
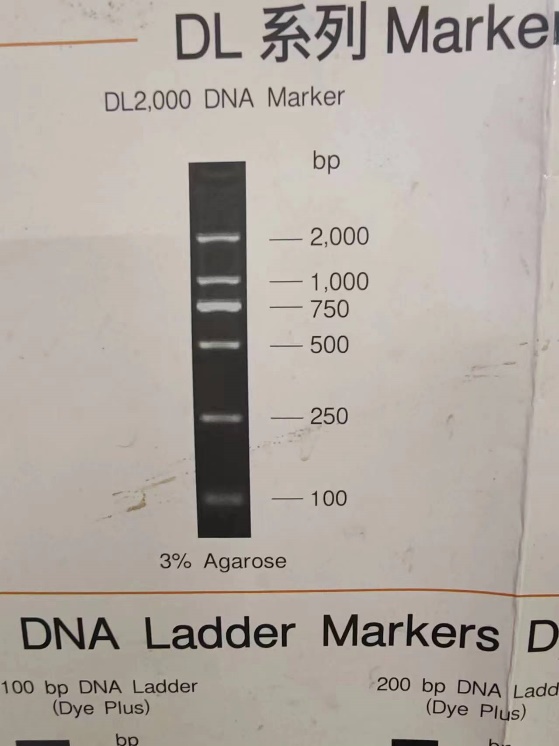
**
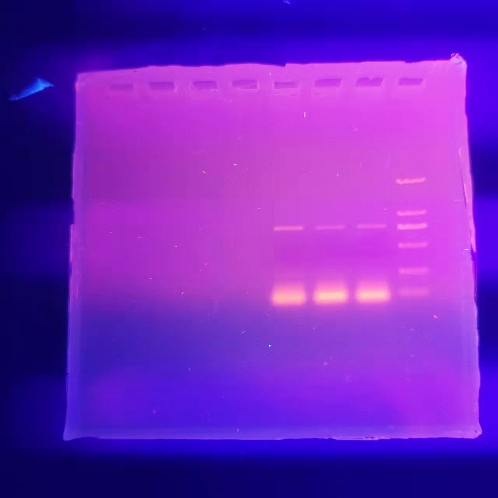


**Figure 5H:** L1, L2: OE-NC; L3, L4: OE-LOC106505926

**Figure 5K:** L1, L2:si -NC; L3, L4: si-LOC106505926

**Figure 6A:** The DNA Maker was shown on the left and the gel was shown on the right. L1, L2, L3: Overexpression vector+CXXC5; L4, L5, L6: Overexpression vector (M: DNA marker L: DNA lanes)

**
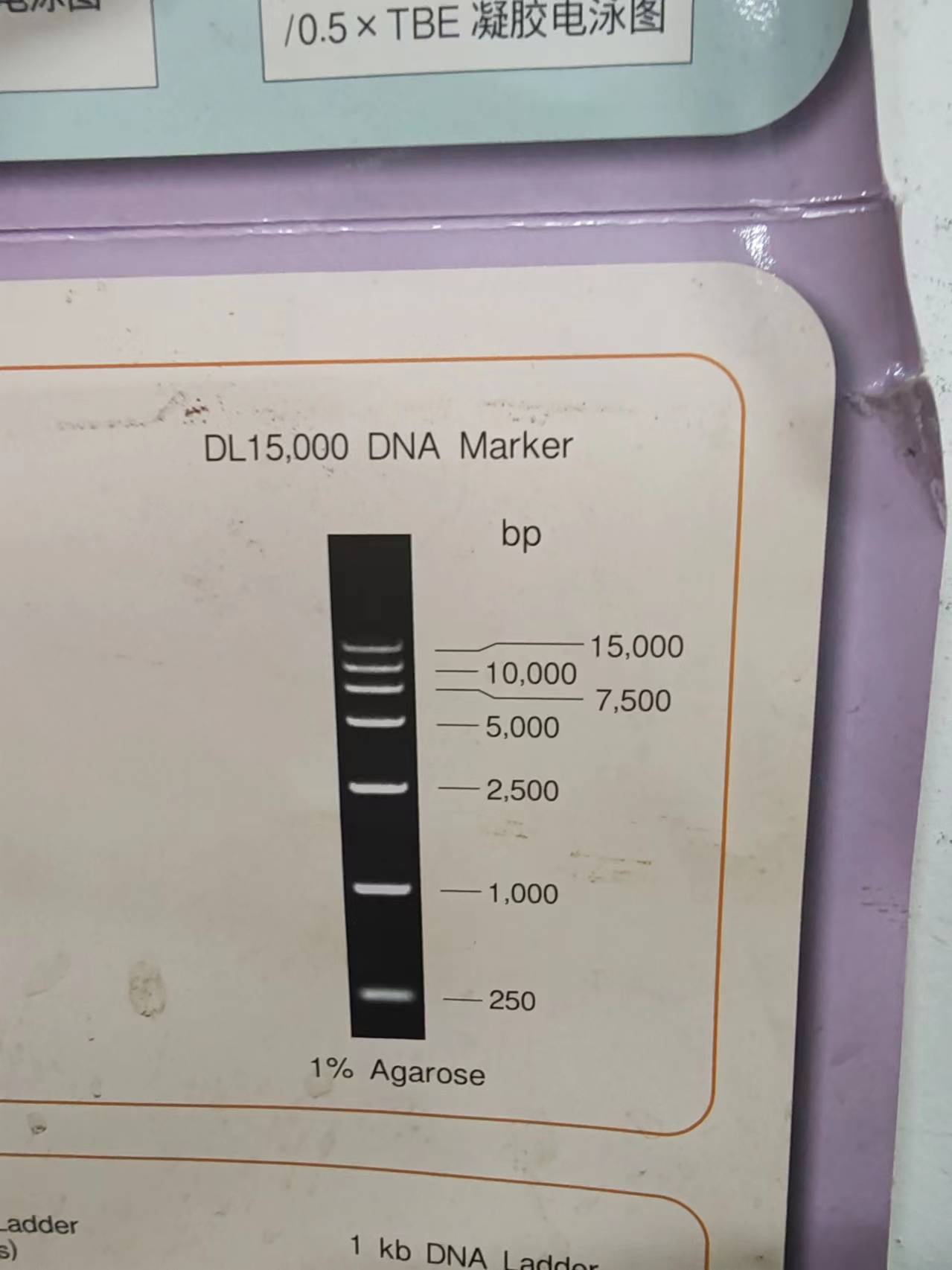
**
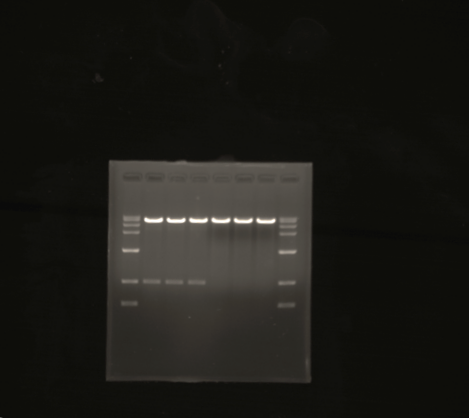


**L5V**

**L6V**

**L4V**

**L3V**

**L2V**

**L1V**

**M**

**M**

**Figure 6B:** L1, L2: OE -NC; L3, L4: OE-CXXC5

**Figure 6J:** L1, L2: OE -NC; L3, L4: OE-CXXC5

**M**

**200 KD**

**Figure 6M:** L1, L2: si -NC; L3, L4: si-CXXC5

**Figure 7A:** L1: NC; L2: OE-CXXC5; L3: OE-CXXC5+miR-22-5p mimic; L4: miR-22-5p mimic

**Figure 7B:** L1: NC; L2: OE-LOC106505926; L3: OE-LOC106505926+miR-22-5p mimic; L4: miR-22-5p mimic

**Figure 8C:** L1,L2: si-NC; L3, L4: si-LOC106505926

**Figure 8F:** L1: Input; L2: Sense; L3: Anti-sense
